# PD14 The Cost Effectiveness Of Robot-Assisted Gait Training For Patients In Japan With Subacute Hemiplegic Stroke

**DOI:** 10.1017/S0266462324002824

**Published:** 2025-01-07

**Authors:** Yasuhiro Morii, Kagari Abiko, Kazuki Ohashi, Toshiya Osanai, Yuji Tani, Takumi Tanikawa, Soichiro Takamiya, Kensuke Fujiwara, Katsuhiko Ogasawara

## Abstract

**Introduction:**

Although it is reported that robot-assisted gait training (RAGT) with conventional physiotherapy for stroke patients increases the odds of walking independence at the end of the intervention, compared with conventional physiotherapy alone, it is not clear how long the benefit lasts or whether it is cost effective. This research aimed to clarify the duration of benefit of RAGT and its cost effectiveness.

**Methods:**

A cost-utility analysis was conducted that compared RAGT plus conventional physiotherapy with conventional physiotherapy alone from a public healthcare payer’s perspective. The population comprised patients with subacute hemiplegic stroke who had a modified Rankin Scale of three to five (severe) and were treated in Japanese hospitals. The time horizon was half a year after admission to a convalescent hospital, since no additional benefit has been proved beyond that point. A decision tree model was used for the analysis. The effect of RAGT on walking independence and the durability of the benefit were estimated based on a literature review of randomized controlled trials (RCTs) and a meta-analysis. Costs and utility values were estimated from the literature.

**Results:**

The literature review identified 14 RCTs. A meta-analysis of RCTs with more than two months’ follow-up after the intervention showed a significantly higher rate of independence in walking for the RAGT group at the end of follow-up (risk ratio 1.52, 95% confidence interval: 1.20, 1.93), whereas there was no significant difference between the groups more than three months after treatment. The incremental quality-adjusted life-years (QALYs) was 0.004 and the incremental cost was -USD287, indicating that RAGT with conventional physiotherapy was dominant. The incremental cost-effectiveness ratio was USD5,509 per QALY when it was assumed that the length of stay was not reduced by achieving early independence in walking.

**Conclusions:**

The results showed excellent cost effectiveness for RAGT plus conventional physiotherapy in patients with subacute severe hemiplegic stroke in the Japanese setting when considering a reference value of USD34,000 (JPY5,000,000) per QALY in the cost-effectiveness evaluation. Although the incremental QALY gains were relatively small, cost savings could be expected from achieving early independence in walking.

